# Participation profiles among Chinese stroke survivors: A latent profile analysis

**DOI:** 10.1371/journal.pone.0244461

**Published:** 2020-12-31

**Authors:** Yuxia Li, Xuemei Li, Lanshu Zhou

**Affiliations:** School of Nursing, Second Military Medical University, Shanghai, China; German Centre for Neurodegenerative Diseases Site Munich: Deutsches Zentrum fur Neurodegenerative Erkrankungen Standort Munchen, GERMANY

## Abstract

**Objectives:**

To investigate the current status of participation and explore the characteristics of individuals with different levels of participation among stroke survivors in mainland China.

**Design:**

Cross-sectional survey.

**Setting:**

Participants were recruited by convenience sampling from the neurology department of the tertiary hospitals and communities.

**Subjects:**

Stroke survivors (N = 517; mean (±SD) age, 69.97±11.51 y; 36.8% female).

**Intervention:**

Not applicable.

**Main outcome measures:**

Participation was measured using the Chinese version of the Impact on Participation and Autonomy Questionnaire (IPA). Rating of disability was assessed using the Modified Rankin Scale (mRS). The Perceived Social Support Scale (PSSS), the Medical Coping Modes Questionnaire (MCMQ), and the Herth Hope Scale (HHS) were also employed to measure social support, coping strategy, and hope of stroke survivors. The latent profiles analysis (LPA) was conducted using the M*plus* version 8.3.

**Results:**

The mean score of participation was 41.21±21.204. Participants were divided into three groups according to the participation using the LPA. The mean score on the sum of IPA for the high, medium, and low participation groups was 18.93±8.529, 42.50±8.302, and 69.44±9.516, respectively.

**Conclusions:**

Stroke survivors have a low level of participation. Stroke survivors with low income, high mRS stage, bad health condition, and being dissatisfied for life tended to have low participation. Healthcare professionals should pay special attention to them and make targeted interventions based on their characteristics.

## Introduction

Stroke is now the third leading cause of disability [1] and the second leading cause of mortality [2] worldwide. Nearly 800,000 individuals suffer from stroke every year, many of whom consequently experience persistent difficulty with daily living [3]. Despite prompt recognition of stroke symptoms, precise diagnosis, and improved systems to deliver care, many patients continue to experience residual functional impairments that have detrimental impacts on both social and physical outcomes [4]. Previously, a considerable body of research has addressed the physical and psychological problems of stroke survivors [5], which contribute to a decrease in the quality of life (QoL) post stroke [6].

Physical problems of stroke survivors are primarily reduced functional independence and deterioration of activities of daily life. Dysfunction is generally regarded as the major factor decreasing the QoL of stroke survivors. Nevertheless, a reduction in QoL is still observed even when no or minimal physical impairment is present [7]. Thus, some studies focusing on social health were conducted in decades.

Participation was first applied in the area of rehabilitation since the year 2001 [8]. According to the guidance of the World Health Organization’s (WHO) latest International Classification of Functioning, Disability and Health (ICF) framework [9], participation means involvement in a life situation, which is related to all aspects of the physical, social, and attitudinal world. Participation includes 1) performing in domains of learning and applying knowledge, 2) doing general tasks and demands, 3) communicating effectively, 4) having mobility, 5) and engaging in self-care, domestic life, interpersonal interactions and relationships, major life areas, community, and social and civic life. Participation restrictions limit the individuals’ involvement in life situations, which can prevent individuals from joining social activities. Increasing participation is currently the primary goal of rehabilitation.

Previously, a considerable body of research has addressed that patients who return to society and participate in social activities can effectively enhance their QoL and reduce the societal and economic burdens of their disease. Social participation was associated with better physical health among the Malawi population, and a better QoL was found in those patients with PD who participated in social activities [10]. A three-year longitudinal study [11] among Japanese older people in urban area showed that social participation could significantly reduce the level of social isolation. Chen’s research determined that participating in social activities was associated with less anxiety among individuals with autism spectrum disorders [12]. A study among stroke survivors and their caregivers demonstrated that social participation heightened the life satisfaction of the stroke-caregiver dyad [13]. It is hard [14] for stroke survivors to return back to the original life. Three focus group interviews among stroke survivors in Sweden [15] found that participants were eager to participate in society and had reintegration practices. Healthcare professionals are supposed to provide them with some guidance to improve their participation abilities and participation willingness [14]. Fortunately, evidence showed that relevant interventions have a positive effect on promoting social participation, which enhances the possibility of more participation [16, 17].

Given the potential effect of participation on improving rehabilitation outcomes, researchers have conducted many studies on participation among the elderly and patients with cancer, spinal cord injury, and other diseases or disabilities [18–21]. However, few studies focus on patients with Chinese stroke survivors. The current status of participation among stroke survivors was not clear. Furthermore, it is impossible to evaluate the participation level of the stroke survivor as there is no authoritative standard for participation among stroke survivors. Only if participation is exactly assessed is it possible to distinguish the stroke survivors who need extra interventions focusing on participation. Consequently, it is essential to establish a personalized and accurate standard of participation among Chinese stroke survivors.

Traditionally, the analytical approaches, such as analysis of variance (ANOVA), multiple regression, focus on relations among variables and assuming the sample under study arises from a homogeneous population [22], namely variable-centered. However, the participants involved in a study are most likely a mixture of different social demography characteristics and disease situation [23], which indicates population heterogeneity.

The mixture modeling (person-centered approach) provides an important complement to the traditional variable-centered analytical approaches [24]. It offers the opportunity for researchers to identify unknown a priori homogeneous groups of individuals based on the measures of interest, examine the features of heterogeneity across the groups, evaluate the effects of covariates on the group membership, and assess the relationship between the group membership and other outcomes. Consequently, the present study will adapt a person-centered approach focusing on identifying unobserved sub-populations comprised of similar individuals or cases to explore the participation of stroke survivors.

Mixture models include the latent class analysis (LCA) model, the latent transition analysis model, the growth mixture model, and the factor mixture model. Classical LCA models use categorical indicators/ items. When continuous indicators/ items are used for clustering, the model is usually called latent profile analysis (LPA). The LPA is employed in this study as the continuous items are used for analyses [25].

Therefore, this present study aimed to investigate the current status of participation, establish the standard of participation, extinguish the characteristics of stroke survivors in need of intervention among stroke survivors in mainland China. The results are intended to provide a reliable and valid measure of perceived participation for stroke survivors, which is essential for the establishment and evaluation of participation interventions and training programs. In addition, this study aimed to investigate the current status of perceived participation in Chinese stroke survivors.

## Materials and methods

### Participants

Participants were recruited through convenience sampling from the Department of Neurology in some tertiary hospitals and communities between March 2019 and June 2019. Inclusion criteria were adult (> = 18 years old) patients who had been diagnosed with stroke according to the standard set by the Chinese Medical Committee [26] and confirmed by magnetic resonance imaging and had the experience of home rehabilitation. The main exclusion criteria were (1) victims of transient ischemic attack; (2) suffering severe organ dysfunctions, respiratory failure, and malignant cancer; (3) having a history of dementia, mental deficiency, and other psychiatric diseases; and (4) having visual, aural, and verbal impairment.

### Procedures

Participants were recruited to the study from the clinic or inpatient ward in the hospital and communities. Every participant was informed about the purpose of the research through face-to-face instructions to assure voluntary participation. Written informed consent was obtained from all participants. Researchers gave instructions about completing the questionnaire, administrated the questionnaire survey, and collected questionnaires immediately following completion. Researchers would read the questions for participants one by one if they cannot read by themselves. The research was approved by the Translational Medicine Ethic Committee, Second Military Medical University (NO. TMEC2018-004).

### Measures

#### Sociodemographic characteristics

Sociodemographic data were collected as part of the research and included gender, age, marital status, education level, monthly income level, medical insurance, degree of stroke knowledge, number of stroke onset, type of stroke, complications of stroke, history of chronic diseases (hypertension, diabetes, and hyperlipidaemia), time of first aid, life satisfaction, and overall health condition.

#### Participation

The original English version of the IPA is a self-report questionnaire consisting of 32 items and five dimensions devised as a Likert-type additive scale with five response choices to assess perceived participation [26]. Participants indicate their degree of possible participation using a 5-point scale ranging from very much (0) to very little (4). Sum scores are calculated per dimension, and total scores are computed to assess respondents’ level of perceived participation; greater scores indicate better participation. The Chinese version of the IPA was first translated and adapted by Li [27]. In the process of revising, the dimension work and education were deleted as most patients were old and had retired. Also; item 6f intimate relationship (sex) was removed out of respect for the Chinese culture [27]. Consequently, the Chinese version of the IPA consists of 25 items and four dimensions: autonomy indoors, autonomy outdoors, family role, and social relations.

#### Rating of disability

The Modified Rankin Scale (mRS) has been used worldwide to evaluate the degree of disability of stroke survivors [28]. Patients were divided into six stages according to their degree of disability: from 0 to 5. The higher the stage, the more severe the disease. The stage 3 means patients are able to walk by themselves through they need some other help. Patients in stage 4 and 5 cannot walk without others’ help.

#### Hope

The Herth Hope Scale (HHS) was employed to assess the level of hope [29]. The HHS was composed of 12 items. And the scale included three dimensions, namely the positive attitudes for the reality and the future, the positive action, and the intimate relationship with others. The score of each dimension was the average score of items included. The higher the score, the higher the level of hope. The Cronbach’s alpha coefficient of the HHS was 0.87 and the test-retest reliability was 0.92, which indicated sound reliability. The construct validity of the scale was 0.85.

#### Social support

The Perceived Social Support Scale (PSSS) is a self-administrated questionnaire composed of 12 items [30]. Degree of agreement is measured using a 7-point Likert scale ranging from *strongly disagree* (1) to *strongly agree* (7). The scale reflects individual understanding and perceived support from family, kin, friends, colleagues, and so on, with higher scores indicating stronger perceived social support. The Chinese version of the PSSS has satisfactory psychometric attributes [31]. Cronbach’s alpha of the scale is 0.91. The test-retest reliability is 0.85.

#### Coping modes

The Medical Coping Modes Questionnaire (MCMQ) is a self-rating scale consisting of 20 items [30]. The MCMQ contains three types of coping strategies (subscale), namely confronce, avoidance, and resignation. Degree of agreement is measured using a 4-point Likert scale ranging from *never* (1) to *always* (4). The score of the subscale confronce is the average score of items 1, 2, 5, 10, 12, 15, 16, and 19. The score of the subscale avoidance is the average score of items 3, 7, 8, 9, 11, 14, and 17. The score of the subscale resignation is the average score of items 4, 6, 13, 18, and 20. The higher the score of a subscale, the more likely the individual tends to adopt this coping strategy. Previous studies showed that the scale has good validity and reliability.

#### Statistical analysis

Data were analyzed using the IBM SPSS version 25.0^a^. Descriptive analyses were initially conducted to characterize the sample. Descriptive statistics for the demographic variables were calculated, including the mean and standard deviation (SD) for continuous variables, such as age, the IPA total and subscale scores, and the total score and each dimension score for the PSSS. Frequency counts and percentages were used to summarize categorical variables, including gender, the mRS stage, marital status, and education level.

The LPA was conducted using the M*plus* version 8.3. The correct number of classes is the key to detecting real population heterogeneity in the LPA [32]. The most common model selection test methods for determining the number of potential categories are mainly two types. The most recommended value for determining the number of potential classes is the value of entropy, which ranges from 0 to 1 [24]. The closer it is to 1, the more accurate the classification. In addition, the fitness of a model is indicated by the value of Bayesian Information Criterion (BIC) and the value of Sample Size-Adjusted BIC (aBIC) [25]. Smaller value indicates better fit. At the same time, Lo-Mendell-Rubin Ajusted LRT (LMR aLRT) is mostly used to compare the fitting differences between the models with k-1 and k classes (k means the number of classes). Significant LMR *P* value means that the model with k classes is better than that with k-1 classes.

## Results

Patients with stroke (n = 517) receiving care in a tertiary hospital in Shanghai, China, completed the questionnaire. Of these participants, 36.8% were female. The mean (±SD) age of the participants was 69.97±11.51 years old, ranging from 32 to 96 years. Two hundred twelve (41.2%) patients had stroke for less than one year and 155 (30.1%) of them had stroke for one to five years. Only 190 (36.8%) of the patients received rehabilitation training after stroke. Table 1 presents the sociodemographic and disease characteristics of these participants.

**Table 1:** Descriptive characteristics of the participants (n = 517).

Demographic Characteristic	n (Percentage)	Disease Characteristic	n (Percentage)
Gender		Type of stroke	
Male	327 (63.2)	Ischemic	462 (89.4)
Female	190 (36.8)	Haemorrhagic	53 (10.2)
Marriage status		Both	2 (0.04)
Married or partnered	443 (85.7)	Function deficits	
Never married, Separated, Divorced, Widowed	74 (14.3)	Having	441 (85.3)
Education level		Not having	76 (14.7)
Elementary school	105 (20.3)	Limb dysfunction	
Middle school	196 (37.9)	Having	302 (58.4)
High school/Vocational Training	134 (25.9)	Not having	215 (41.6)
College or above	82 (15.9)	Rehabilitation training	
Monthly income (¥)		Yes	190 (36.8)
< 1000 RMB	6 (1.16)	No	327 (63.2)
1000~2999 RMB	87 (16.83)	Stroke Knowledge	
3000~4999 RMB	262 (50.68)	None	142 (27.5)
≥5000 RMB	162 (31.33)	Some	325 (62.9)
Working status		Very much	50 (9.7)
Employed	47 (9.1)	Modified Rankin Scale stage	
Retired/Unemployed	470 (90.9)	0	28 (5.4)
Living arrangement		1	162 (31.4)
Alone	41 (7.9)	2	142 (27.5)
With spouse	425 (82.2)	3	77 (14.9)
With others	51 (9.9)	4	90 (17.4)
		5	17 (3.3)

RMB: Ren Min Bi (the Chinese yuan, ¥).

The monthly family income of patients was usually more than 3000 RMB (n = 424, 82.0%) and 425 (82.2%) patients lived with their families. Most patients (n = 503, 97.3%) had medical insurance. Three hundred fifty-four patients (68.5%) were victims of first-time stroke. A majority of patients (n = 462, 89.4%) suffered ischemic stroke. The number of patients who suffered complications of function deficits and limb dysfunction were 441 (85.3%) and 302 (58.4%), respectively. Most (n = 410, 79.3%) participants were satisfied with their life.

### Descriptive statistics

The mean score on the sum of the IPA among all participants was 41.21±21.204. The mean scores on the four dimensions (autonomy indoors, family role, autonomy outdoors, and social relations) of the IPA were 1.15±1.11, 2.01±1.14, 1.14±0.70, and 2.24±0.99, respectively.

### Latent profiles analysis

The LPA was conducted taking the 25 items of the IPA as the indicators. The input statement of the LPA was shown in S1 Table (taking the three-class for example).

Table 2 demonstrated the model fit information for different models. The value of indicates that the three-class model is the best model. The value of entropy shows that four models are all acceptable. The five-class model has the smallest value for AIC and BIC. Taking the three factors into account, the three-class model is the most ideal. In addition, the distribution of case number and class probability in the three-class model is much more reasonable than others. Table 3 shows the details of probability and case number of different classes in the LPA.

**Table 2:** Model fit information for different models.

Models	AIC	BIC	aBIC	LMR aLRT	BLRT	Entropy
				Test *P*	*P*	
1-class	39947.969	40160.371	40001.661	—	—	—
2-class	33780.739	34103.590	33862.351	0.0023	0.0024	0.973
3-class	31508.614	31941.914	31618.147	0.0043	0.0045	0.968
4-class	30280.751	30824.501	30418.204	0.3886	0.3906	0.964
5-class	29587.730	30241.928	29753.103	0.3652	0.3677	0.974

Notes: AIC, Akaike Information Criterion; BIC, Bayesian Information Criterion; aBIC, Sample Size-Adjusted BIC; LMR aLRT, Lo-Mendell-Rubin, Ajusted LRT; BLRT, Bootstrapped Likelihood Ratio Test.

**Table 3:** Probability and case number of different categories in the latent profile analysis.

Model	Category probability	Case number
1-class	—	—
2-class	0.64/0.36	331/186
3-class	0.35/0.40/0.25	178/208/131
4-class	0.28/0.23/0.40 /0.09	144/120/206/47
5-class	0.09/0.31/0.32/0.19/0.09	51/159/165/98/44

**Fig 1:**
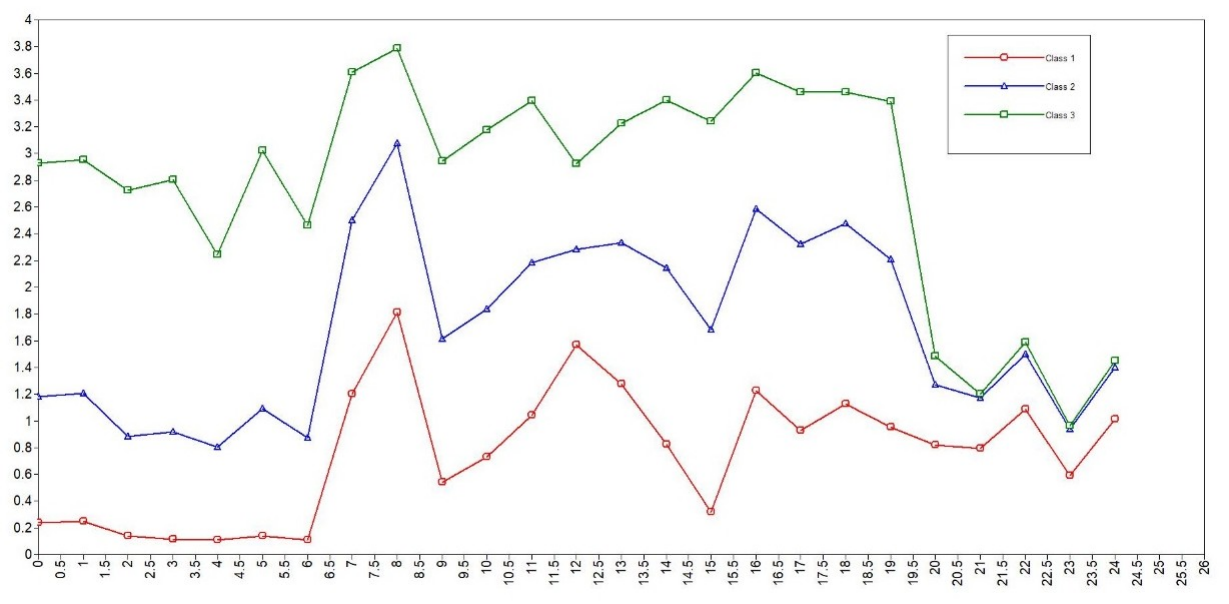
The distribution of three potential classes with different participation levels.

The participants were then divided into three groups based on the results of the LPA according to the level of participation, namely group of high participation (n = 178), group of medium participation (n = 208), and group of low participation (n = 131). Fig 1 shows the distribution of three potential classes with different participation levels. The mean score on the sum of IPA for the high, medium, and low participation groups was 18.93±8.529, 42.50±8.302, and 69.44±9.516, respectively. The mean scores on the four dimensions (autonomy indoors, family role, autonomy outdoors, and social relations) were 0.158±0.30, 0.84±0.55, 0.86±0.66, and 1.27±0.66 for the high participation group, 1.00±0.45, 2.15±0.63, 1.26±0.68, and 2.37±0.58 for the medium participation group, and 2.73±0.71, 3.36±0.58, 1.33±0.67, and 3.35±0.48 for the low participation group. The differences on the scores of four dimensions between every two groups are all statistically significant.

Characteristics of individuals in three groups are also different, including social demography information and disease situation. The difference on the composition of the variables below were statistically significant: monthly income, stroke knowledge, number of years after stroke, life satisfaction, health condition, and mRS. Stroke survivors showing higher level participation had enough knowledge of stroke, more monthly income, lower mRS stage, and better health condition and felt much more satisfied for life. Individuals in the group of high participation showed a higher level of hope, more social support, and tended to adapt the coping strategy of confronce. Table 4 shows the characteristics of individuals in different groups in detail.

**Table 4:** Characteristics of individuals in different potential groups based on participation level by the latent profile analysis (n = 517).

Variables/Groups	High level (n = 178) n (%)/score	Medium level (n = 208) n(%)/score	Low level (n = 131) n (%)/score	χ^2^	*P* value
Monthly income (¥, RMB)				14.599	0.024*
< 1000	2(1.1)	2(1.0)	**2(1.5)**		
1000~2999	18(10.1)	41(19.7)	**28(21.4)**		
3000~4999	87(48.9)	**111(53.4)**	64(48.9)		
≥5000	**71(39.9)**	54(26.0)	37(28.2)		
Working status				6.769	0.034*
At work	254(92.4)	88(94.6)	**128(85.9)**		
Off work	21(7.6)	5(5.4)	21(14.1)		
Stroke Knowledge				15.314	0.004**
None	35(19.7)	64(30.8)	**43(32.8)**		
Some	**116(65.2)**	128(61.5)	81(61.8)		
Very much	**27(15.2)**	16(7.7)	7(5.3)		
Number of years after stroke				14.895	0.021*
< 1	79(44.4)	72(34.8)	**61(46.9)**		
1~5	56(31.5)	**71(34.3)**	28(21.5)		
5~10	**27(15.2)**	27(13.0)	17(13.1)		
> 10	16(9.0)	37(17.9)	**24(18.5)**		
Life satisfaction				137.595	<0.001**
Very Dissatisfied	0(0)	4(1.9)	**18(13.7)**		
Dissatisfied	14(7.9)	29(13.9)	**42(32.1)**		
Neutral	48(27.0)	**116(55.8)**	42(32.1)		
Satisfied	**98(55.1)**	54(26.0)	22(16.8)		
Very satisfied	**18(10.1)**	5(2.4)	7(5.3)		
Health condition				152.278	<0.001**
Very bad	0(0)	4(1.9)	**10(7.6)**		
Bad	13(7.3)	44(21.2)	**65(49.6)**		
General	88(49.4)	**132(63.5)**	51 (38.9)		
Good	**72(40.4)**	28(13.5)	5(3.8)		
Very good	**5(2.8)**	0(0)	0(0)		
Modified Rankin Scale stage				506.222	<0.001**
0	**26(14.6)**	1(0.5)	1(0.8)		
1	**119(66.9)**	43(20.8)	0(0)		
2	30(16.9)	**108(52.2)**	4(3.1)		
3	3(1.7)	43(20.8)	**31(23.7)**		
4	0(0)	12(5.8)	**78(59.5)**		
5	0(0)	0(0)	**17(13.0)**		
Hope	**35.04±3.653**	33.65±3.113	33.31±3.942	11.298	<0.001**
Social support	**59.00±10.794**	55.06±10.230	54.36±10.768	9.455	<0.001**
Coping strategy					
Confronce	**2.18±0.585**	2.00±0.539	1.87±0.602	11.466	<0.001**
Avoidance	2.19±0.353	**2.31±0.337**	2.23±0.357	5.480	0.004**
Resignation	1.63±0.554	2.04±0.579	**2.39±0.706**	59.863	<0.001**

Notes: RMB, Ren Min Bi (the Chinese yuan, ¥);

* *P*<0.05

** *P*<0.01.

## Discussion

The present study investigated the current status of participation and extinguished the characteristics of stroke survivors in need of intervention among stroke survivors in mainland China. The result of this study provided a scientific standard of high, medium, and low participation. In addition, this study helps the healthcare providers find out the stroke survivors in need of intervention and make targeted interventions based on the characteristics of the certain population in a low level of participation.

First of all, this study investigated the current status of participation among Chinese mainland stroke survivors. The mean score on the IPA among stroke survivors was a bit higher (*t* = 4.107, *P*<0.05) than the 40.39±15.29 from the research of Chen and colleagues on stroke survivors that had a similar age (67.9±12.5Y) and lower monthly income (≤3000 RMB, 57.2%) [33], which indicated a worse participation. Consequently, it is necessary to take some measures to promote the participation of stroke survivors and ultimately improve their rehabilitation outcomes.

Secondly, the present study established a scientific standard to evaluate the level of participation for stroke survivors in mainland China. The standard for high, medium, and low level of participation on the sum score of the IPA among stroke survivors is 0~29.9, 30.0~54.9, and 55.0~100, respectively. When measuring the participation of a stroke patient, the researcher can accurately define his/her level of participation. Then healthcare providers can pay attention to stroke survivors with low level of participation and provide targeted interventions for them. The cut points of different levels are essentials for evaluating and determining whether extra interventions are needed. The results can to some extent provide practical references for future studies on participation among stroke survivors.

Thirdly, the results of this study demonstrated the characteristics of the stroke survivors with a low level of participation. In other words, the results provided relatively accurate information for health care providers to find out the stroke survivors in need of intervention. Stroke survivors with low monthly income, off work, insufficient knowledge about stroke, bad health condition, and high mRS stage tended to have a low level of participation. The mRS stage represents the degree of disability of stroke survivors. To some extent, the level of participation can be predicted by the severity of stroke. Besides, the higher the mRS stage, the more barriers the patients have on the process of taking part in various activities. It is easy to understand that patients having higher income and with enough knowledge about the disease showed better participation as they known more about their own disease and had sufficient income to engage in more activities. In addition, patients with high levels of knowledge understand that the rehabilitation can improve the stroke outcome and persistent treatment and rehabilitation exercise will help them. Social interactions at work allowed participants to forget a little about stroke related impairments and had more opportunities to participate in society. On one hand, this suggests that healthcare professionals should pay special attention to the patients with low monthly income, off work, insufficient knowledge about stroke, bad health condition, or high mRS stage. On the other hand, interventions for stroke survivors with low level of participation should include general health education, stroke rehabilitation guidance, and specific intervention focusing on participation abilities and willingness.

Last but not the least, this study may provide a theoretical basis for the formulation of intervention measures focusing on participation for stroke survivors. Stroke survivors in different groups have different characteristics and appropriate interventions are supposed to apply to each group. Take the group with low level of participation for example, stroke survivors in this group showed relatively severe disability, low level of hope, and resignation style of coping. Consequently, interventions for stroke survivors in this group should include both rehabilitation guidance and specific intervention focusing on participation abilities and willingness. Stroke survivors with high level of participation showed high level of hope, and confronce style of coping, which indicated that hope and confronce style might be facilities of participation. This provided a new scope for health professionals to enhance the level of participation. Targeted interventions are supposed to emphasize on the health education about stroke knowledge, change in coping strategy, perception on social support, and hope for the future.

### Study limitations

There are several limitations in this study. Firstly, this study was conducted in one city in China, which may cause sampling bias due to the differences in diagnosis, treatment, and rehabilitation levels between diverse provinces or regions. Stroke survivors from more regions should be included in future studies. Secondly, most patients involved in the present study suffered from a mRS stage between 0 and 3. Patients with a mRS stage higher than three often suffer from severe disability and would not be willing to complete this survey. Actually, it is very difficult for them to return the normal social life as they cannot walk independently. It is more urgent to have rehabilitation training for stroke survivors on the mRS stage 4–5. Consequently, the present study may reflect the participation status of mild to moderate stroke survivors in China. We will focus on the participation of the stroke survivors with relatively severe disability to improve their quality of life.

Despite these limitations, the study provides new information on the current status of participation among Chinese stroke survivors, which is the basic of further accurate assessment and targeted intervention formulation. This could contribute to the promotion of participation among Chinese stroke survivors.

### Conclusion

This study focusing on the participation among Chinese stroke patients demonstrated that the participation level of Chinese stroke survivors was not optimal and various variables were related with participation. Further studies should be done to explore the influencing factors of participation among stroke survivors. Tailored interventions focusing on changeable factors should be implemented in different rehabilitation phases to enhance the participation of stroke survivors in all areas of daily life.

## Supporting information

S1 Table The input statement of the latent profile analysis (take the three-category for example). (PDF)
