# Supplementary material for: Participation profiles among Chinese stroke survivors: A latent profile analysis
Source: PLoS One. 2020 Dec 31;15(12):e0244461. doi: 10.1371/journal.pone.0244461 (PMC7774852; doi:10.1371/journal.pone.0244461)
Supplement: S1 Table — (PDF) [file pone.0244461.s001.pdf]

S1 Table The input statement of the Latent Profile Analysis

（take the three-category for example）

```
TITLE:    LPA with P
   DATA: FILE IS C:\Users\lyx\Desktop\P.dat;
             FORMAT IS F3.0 25F2.0;
   VARIABLE:       NAMES ARE ID P1-P25;
            USEVARIABLES ARE P1-P25;
            CLASSES = c (3);
               MISSING=ALL(-1);
   ANALYSIS:       TYPE = MIXTURE;
   OUTPUT:    SAMPSTAT STANDARDIZED TECH11;
   SAVEDATA:SAVE=CPROB;
                 File is P_LCAc3.txt;
                 PLOT:TYPE=PLOT3;
                 SERIES=P1-P25(*);
```
